# Paradigmatic Approach to Support Personalized Counseling With Digital Health (iKNOW)

**DOI:** 10.2196/41179

**Published:** 2023-04-21

**Authors:** Dorothee Speiser, Maren Heibges, Laura Besch, Caren Hilger, Marie Keinert, Katharina Klein, Gudrun Rauwolf, Christine Schmid, Sven Schulz-Niethammer, Steffi Stegen, Viola Westfal, Isabell Witzel, Benedikt Zang, Friederike Kendel, Markus A Feufel

**Affiliations:** 1 Department of Gynecology with Breast Center, Hereditary Breast and Ovarian Cancer Center Charité – Universitätsmedizin Berlin Berlin Germany; 2 Division of Ergonomics Department of Psychology and Ergonomics Technische Universität Berlin Berlin Germany; 3 Gender in Medicine Charité – Universitätsmedizin Berlin Berlin Germany; 4 Department of Clinical Psychology and Psychotherapy Friedrich-Alexander-Universität Erlangen-Nürnberg Erlangen-Nuernberg Germany; 5 BRCA-Netzwerk e.V. Hilfe bei familiären Krebserkrankungen Bonn Germany; 6 Department of Gynecology, Hereditary Breast and Ovarian Cancer Center University Medical Center Hamburg-Eppendorf Hamburg Germany

**Keywords:** hereditary breast and ovarian cancer, BRCA, genetic counseling, digital health, online counseling tool, user-centered design principles, risk communication, cancer risk, hereditary cancer, breast cancer, ovarian cancer

## Abstract

iKNOW is the first evidence-based digital tool to support personalized counseling for women in Germany with a hereditary cancer risk. The counseling tool is designed for carriers of pathogenic gBRCA (germline breast cancer gene) variants that increase the lifetime risk of breast and ovarian cancer. Carriers of pathogenic variants are confronted with complex, individualized risk information, and physicians must be able to convey this information in a comprehensible way to enable preference-sensitive health decisions. In this paper, we elaborate on the clinical, regulatory, and practical premises of personalized counseling in Germany. By operationalizing these premises, we formulate 5 design principles that, we suggest, are specific enough to develop a digital tool (eg, iKNOW), yet wide-ranging enough to inform the development of counseling tools for personalized medicine more generally: (1) digital counseling tools should implement the current standard of care (eg, based on guidelines); (2) digital counseling tools should help to both standardize and personalize the counseling process (eg, by enabling the preference-sensitive selection of counseling contents from a common information base); (3) digital counseling tools should make complex information easy to access both cognitively (eg, by using evidenced-based risk communication formats) and technically (eg, by means of responsive design for various devices); (4) digital counseling tools should respect the counselee’s data privacy rights (eg, through strict pseudonymization and opt-in consent); and (5) digital counseling tools should be systematically and iteratively evaluated with the users in mind (eg, using formative prototype testing to ensure a user-centric design and a summative multicenter, randomized controlled trial). On the basis of these paradigmatic design principles, we hope that iKNOW can serve as a blueprint for the development of more digital innovations to support personalized counseling approaches in cancer medicine.

## Introduction

In recent years, approaches to personalized medicine have elevated the relevance of the genetic causes of cancer for both diagnosis and targeted therapy. The fast-growing knowledge base in this field has the potential to benefit more and more patients with cancer and healthy counselees with a hereditary risk of developing cancer [1]. To realize this potential, physicians must be able to convey up-to-date evidence and complex risk information while tailoring their recommendations for screening programs or preventive surgery to individual needs and preferences. In this paper, we elaborate on how digital technology may be used to support this extensive counseling process and help realize the potential of personalized medicine. We report on the evidence-based design [2] of a digital counseling tool––the iKNOW tool––and, based on it, outline 5 general design principles for digital tools aimed at supporting personalized counseling processes for carriers of pathogenic genetic germline variants and their counseling physicians.

The iKNOW tool is designed to support counseling for 2 of the most common genetically associated cancers: breast cancer and ovarian cancer. At least 5%-10% of the diagnosed cases with breast and ovarian cancer result from pathogenic genetic variants in risk genes [3]. Today, pathogenic variants in the high-risk genes, breast cancer gene 1 (*BRCA1*) and breast cancer gene 2 (*BRCA2*), which were first described in 1992-1994, are particularly well known [4,5]. The carriers of pathogenic variants in these genes are confronted with complex risk information and potentially life-changing health decisions, which may impact the psychosocial well-being of entire families. Given the complexity of a pathogenic variant in *BRCA1* or *BRCA2* and its potential impact on the lives of patients and their families, there is no one best care pathway. Instead, physicians must share the decision-making process and discuss with their patients what option to focus on, how to evaluate the available evidence, and what decision to make based on the patient’s preferences [6]. In the following, we first present the clinical, regulatory, and practical premises, then we show how they informed the development of the iKNOW tool for the counseling of gBRCA (germline breast cancer gene) mutation carriers in Germany, and, finally, we highlight 5 paradigmatic design principles for the digitalization of personalized counseling in general.

## Premises

### Requirements of Personalized Counseling for Hereditary Cancer in Germany

In Germany, both healthy counselees and patients with breast and ovarian cancer with pathogenic gBRCA variants receive specialized counseling and diagnostics in 23 centers of the German Consortium of Hereditary Breast and Ovarian Cancer (GC-HBOC). To tailor risk management strategies to the personal needs and preferences of counselees and patients, a complex array of *individual clinical characteristics* needs to be considered by the counseling physician, such as the exact type of pathogenic variant (eg, *BRCA1*, *BRCA2*), family history, health status, or, in the case of pre-existing disease, the exact tumor biology and stage of the disease [7]. In addition to individual clinical characteristics, *counselees’ personal preferences* and *information needs* must be respected when presenting and discussing the available risk management options, such as intensified screening programs or prophylactic surgery [8]. In Germany, people with genetic conditions have the legally mandated “right to *not* know” (Recht auf Nichtwissen) about certain aspects or consequences of their genetic condition. Thus, physicians must gauge about which aspects and how detailed their patients want to be informed before providing counseling.

The basis of any counseling session for people with a familial cancer history is their individual risk to get cancer and how this risk develops over the years, which can be calculated with validated algorithms such as the Breast and Ovarian Analysis of Disease Incidence and Carrier Estimation Algorithm (BOADICEA) [9]. From a clinical perspective, the individual 10-year risk, that is, the probability that a person will develop a specific type of cancer within the next 10 years, is of central importance to personalized risk management. Unfortunately, our research shows that, among 111 carriers of pathogenic gBRCA variants, 22.6% underestimated and 53.2% overestimated their calculated 10-year risk of developing breast cancer [7]. Therefore, a major challenge for counseling physicians is to translate complicated probabilities for different time periods into easy-to-understand risk formats as a prerequisite for shared decision-making.

A second important ingredient of any counseling session is up-to-date, evidence-based information on screening and preventive measures based on current guidelines. In this context, scientific progress and a *rapidly changing evidence base* are major challenges. This requires each individual physician to keep up with the developing evidence and patient information materials to be updated and replaced iteratively. Finally, the best practice of personalized, preference-sensitive counseling requires physicians to adjust risk management in light of personal circumstances (eg, competing risks of already existing diseases or family planning), the psychosocial well-being of the counselees and their family, and changing personal preferences (eg, the “right to *not* know”) as part of a shared decision-making process [6].

### New Possibilities Through Digital Health Technologies

The key challenges of personalized counseling are, as just outlined, to match counseling contents to individual clinical characteristics, to understand and address the counselee’s personal preferences and information needs, and to keep up with the rapidly changing scientific evidence base. In light of these challenges*, digital health technologies* offer new possibilities to personalize, standardize, update, and thereby improve the counseling process. In particular, information in databases can be easily searched and filtered to identify contents and match them to individual characteristics. Similarly, information can be saved and may be provided in multiple formats and at various levels of detail to (1) help physicians select the information that meets their counselees’ personal preferences and needs and (2) allow counselees to reaccess this personalized information any time after their actual counseling session. Also, evidence updates may be centralized to help unburden busy health professionals. This way, physicians may spend more time on their patients and less time on searching and parsing the latest evidence in their field [10].

In recent years, several web-based counseling tools for people with a family history of breast and ovarian cancer have been developed, especially in English-speaking countries [11]. Research on these tools has shown that they can reduce disease-specific anxiety [12] and increase patients’ satisfaction with their decision-making [13]. Unlike the iKNOW tool, however, they are mainly designed for carriers of g*BRCA1/2* *variants* to explore cancer risks over time [14], weigh decision options [15], or both [16] with or without a physician. To digitally support personalized counseling as a *shared decision-making process*, however, we think that clinical support systems should be designed to support the *interaction* and the information exchange *between* physicians and patients, rather than the individual decision-making process of patients *or* health professionals alone. iKNOW is therefore conceptualized as a *counseling* support tool. Also, to reduce the cognitive complexity during counseling, a digital support tool should ideally document the information exchanged and make it available after the counseling session.

### Personalized Counseling During and After the Consultation

Key to supporting personalized counseling as a shared decision-making process is that information adheres to evidence-based standards, that the available information is tailored to the particular information needs of the counselee (including the “right to *not* know”), and that the counselee has sufficient time to think about the evidence and come to a decision [6]. To do so, we contend that 2 versions of a counseling tool are necessary: a *physician-based* version to be used by the counseling physician and the counselee *during* a consultation and a *patient-based* version for the counselee to be accessed with more time *after* the consultation. During counseling, counselees and physicians can use the tool to decide together which categories of information to discuss based on a database filled with up-to-date evidence-based information about familial cancer. To implement their “right to *not* know,” counselees can also decide which information should be activated so that they may or may not reaccess it with more time and in more detail after the consultation. As a result, the second version of the tool––the patients’ personalized version––contains a documentation of all information that has been discussed during counseling, which can be accessed any time after consultation. Whereas physicians can use this as part of a medical record, patients and counselees may use it to look up information and share the contents with significant others and family members. Should counselees require additional information, they may use the tool to contact the physician for follow-up appointments. This 2-fold structure prevents counselees and patients from feeling overwhelmed by too much information or encountering irrelevant, invalid, or questionable information, which may cause unnecessary worry or fear and, ultimately, overtreatment [17].

## Design Principles Supporting Personalized Counseling With Digital Health

### Presentation of Design Principles

To our knowledge, iKNOW is the first digital tool to support counseling on hereditary cancer in German-speaking countries that supports physicians *and* patients during and after counseling so that they can truly share decision-making. On the basis of the 3 premises discussed above and encouraged by the evidence-based design approach [2], we operationalized the following five design principles: (1) implementing the current standard of care, (2) supporting personalization *and* standardization of the counseling process, (3) making complex information easy to access, (4) respecting counselees’ data privacy rights, and (5) systematic and iterative evaluation with the users in mind. These design principles are specific enough to elaborate the development of a counseling tool such as iKNOW, but they are also general enough to inform the development of digital innovations aimed at supporting personalized counseling in cancer medicine more generally. We present and discuss each principle below.

### Principle 1: Implementing the Current Standard of Care

The core of iKNOW is a database that reflects the standard of care as it is based on the current guidelines and the available evidence. Given that iKNOW was developed for the German health care system, the clinical evidence reflects the current S3 guideline regarding gynecological cancers, in particular, breast cancer and ovarian cancer [18,19]. All processes surrounding the counseling session, for instance, the genetic analysis, follow-up appointments, and the intensified screening program, reflect the standard operating procedures defined in the guidelines of the GC-HBOC [20]. To provide additional patient-relevant information (eg, related to psychosocial support or possible lifestyle changes), information materials of the German Cancer Information Centre [21], the German Cancer Aid [22], and the BRCA network [23] were summarized and the information was referenced in the iKNOW database. Additionally, several workshops and focus group interviews with expert patients from the BRCA network were held to optimally include the patients’ perspectives in the development of the tool.

Given the dynamic developments in the field of hereditary cancers, one feature of the iKNOW database is that it can be continuously updated to reflect the newest evidence and the most recent changes to the guidelines. Thanks to the centralized updating process in the database, physicians can readily access the most up-to-date information and spend their time compiling the evidence that is most relevant to their counselees and identifying a personalized risk management strategy by discussing the evidence with them, rather than having to search for the latest evidence themselves.

### Principle 2: Supporting Personalization and Standardization of the Counseling Process

Counseling on hereditary breast and ovarian cancer requires specialized knowledge on a plethora of different topics (eg, genetics; expert knowledge on gynecologic oncology and breast cancer, both from a surgical and systemic therapy view; fertility counseling; screening options; and psychosocial topics), the ability to personalize that knowledge in order to meet the requirements and preferences of a particular counselee, and a considerable amount of practice to convey all relevant information during a 60-minute counseling session. Counseling tools should therefore structure the information available in the database in a way that supports both the standardization *and* personalization of the available information during counseling to, ultimately, decrease unwanted variation (eg, due to different levels of counseling experience among physicians) and increase the quality of personalized counseling. In the following, we describe the structure we implemented in the iKNOW tool to realize this principle.

To achieve standardization and personalization, iKNOW divides the counseling content into four main categories, which are presented throughout the counseling process in the navigation menu: (1) “Pedigree and risk presentation,” (2) “Genetics and family,” (3) “Screening and prevention,” and (4) “Support and lifestyle.” Each of these main categories contains several subcategories. This organizational scheme outlines the range of the content areas that should be considered during every counseling session, unburdening the physician’s working memory. At the same time, the iKNOW data structure does not constrain the exact topic, the order in which it should be discussed, or whether it should be discussed at all given a counselee’s preferences. Thus, physicians can navigate the contents based on the individual preferences of the counselee without forgetting to address any potentially important information category.

**Figure 1:**
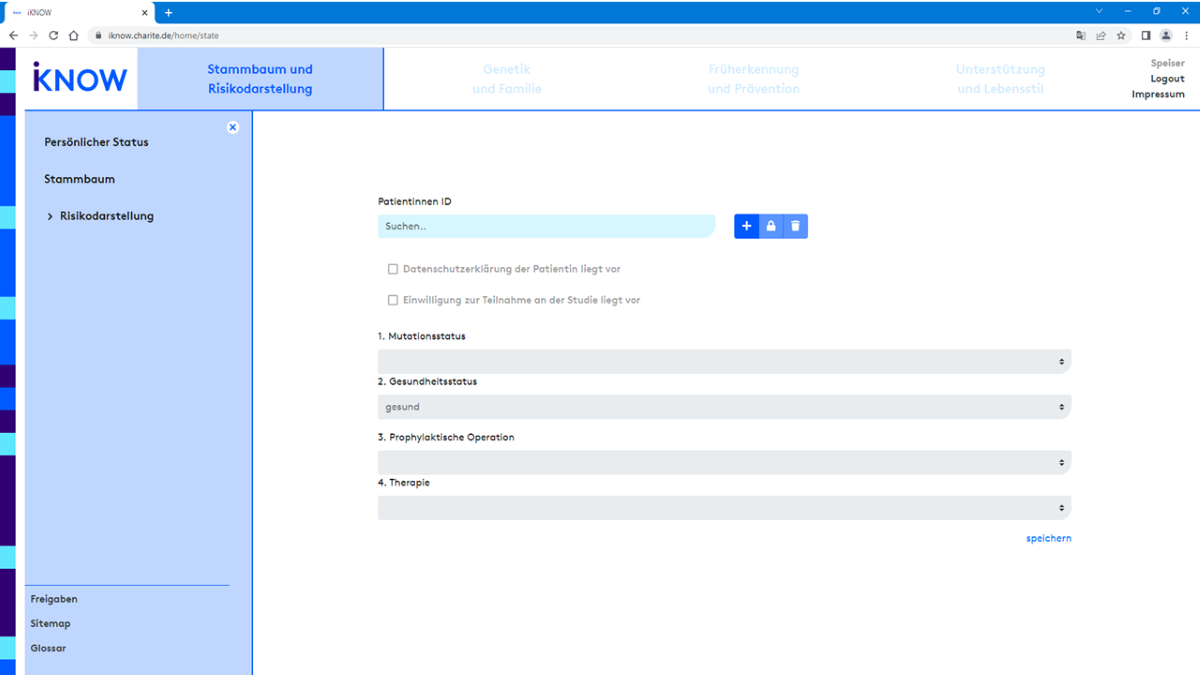
iKNOW counseling tool. At the beginning of each session, the counseling physician may filter the available information with respect to four variables: (1) pathogenic variant (Mutationsstatus), (2) health status (Gesundheitsstatus), (3) prophylactic surgeries (Prophylaktische Operation), and (4) treatment status (Therapie).

To personalize the contents of the counseling session, physicians may further use four filter variables with a defined set of parameter settings at the beginning of each session to compile a subset of the information available in the iKNOW database that is tailored to the clinical situation of the individual counselee: (1) *pathogenic variant* (*BRCA1* or *BRCA2*), (2) *health status* (no cancer, unilateral breast cancer, bilateral breast cancer, ovarian cancer, or metastasized disease), (3) *prophylactic surgeries* undertaken (risk-reducing salpingo-oophorectomy, risk-reducing mastectomy, or no surgery), and (4) *treatment status* (ongoing or finished) (see Figure 1). The number and parameter settings reflect the current gold standard stated in the guidelines. They may be amended to match ongoing changes in the guideline or in the evidence base.

### Principle 3: Making Complex Information Easy to Access

**Figure 2:**
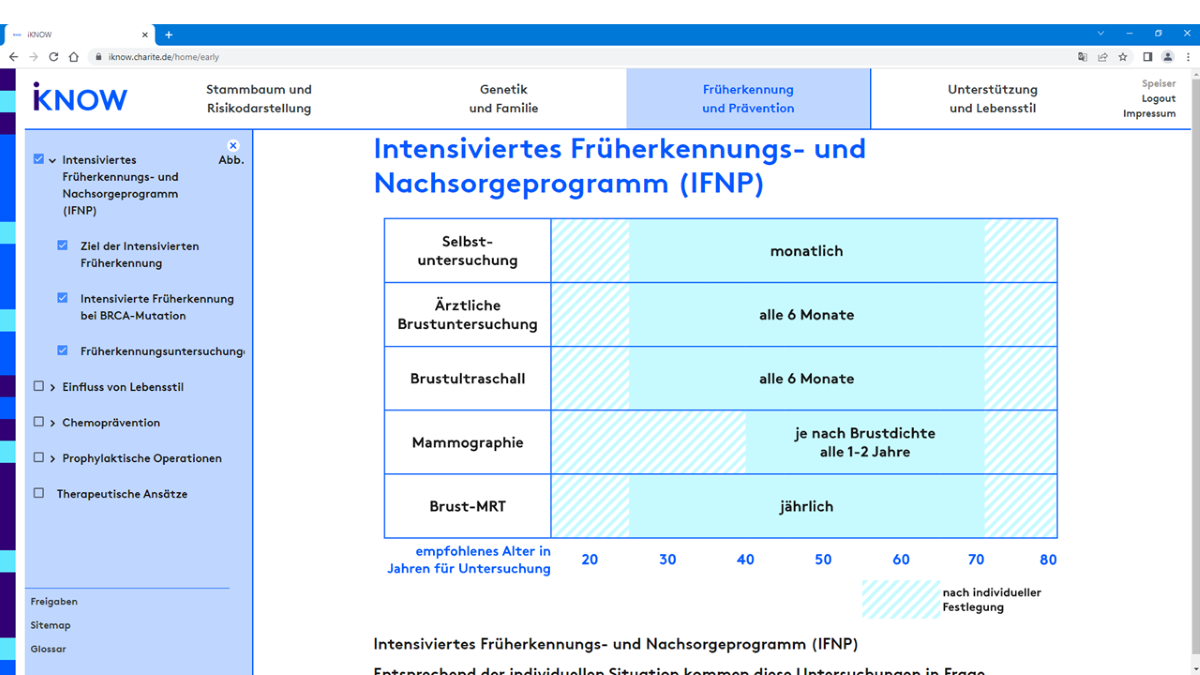
iKNOW counseling tool. Visual representation of information in the physician’s version used during counseling (here: to convey information about the recommended appointments in the intensified screening program for carriers of pathogenic germline breast cancer gene [gBRCA] variants in Germany). MRT: MRI (magnetic resonance imaging).

**Figure 3:**
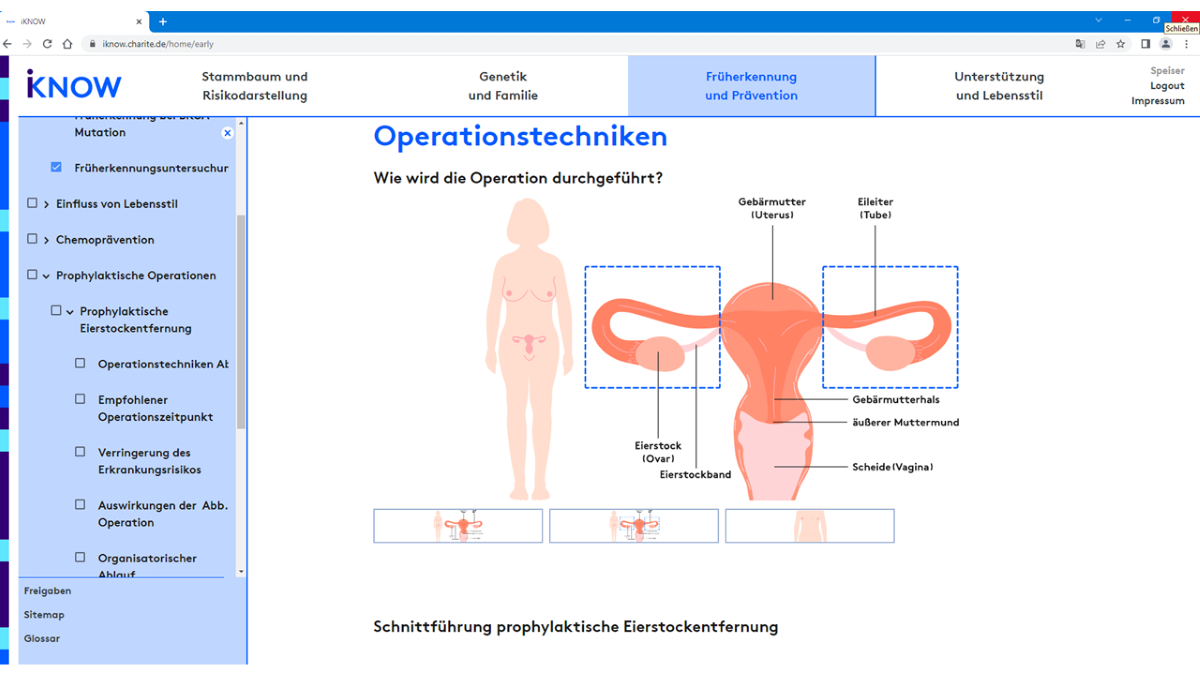
iKNOW counseling tool. Schematic representation of pelvic organs and surgical access during risk-reducing salpingo-oophorectomy, a type of operation in which a surgeon removes the ovaries and fallopian tubes.

To support the counseling process, a central goal of any digital support tool should be to make complex medical and risk information easier to access both cognitively (ie, in terms of tool usability and comprehensibility of information contents) and technically (ie, in terms of accessibility via responsive interfaces for different devices). Importantly, user testing during the development process of iKNOW suggested that too much text and too many visuals on the computer screen during counseling may attract attention away from the doctor-patient interaction. On the other hand, explanatory text is beneficial for patients who want to *follow up* on their counseling session with additional reading after the consultation. Thus, explanatory text should be available *after* counseling but used sparingly *during* the session and so should tables and visualizations. When the iKNOW tool is used during a counseling session, tables and visualizations are thus used mainly to explain complex medical relations (eg, genetic inheritance, the intensified screening, and prevention program) and to create a shared understanding of these issues between physicians and counselees (eg, Figure 2). This context-sensitive approach to information provision is reflected in the adaptive information structure of iKNOW, which comprises three different levels of information: (1) only headings; (2) headings plus visual representations such as tables (see Figure 2), flowcharts, and schematic drawings (see Figure 3); and (3) headings plus visual representations plus explanatory text.

**Figure 4:**
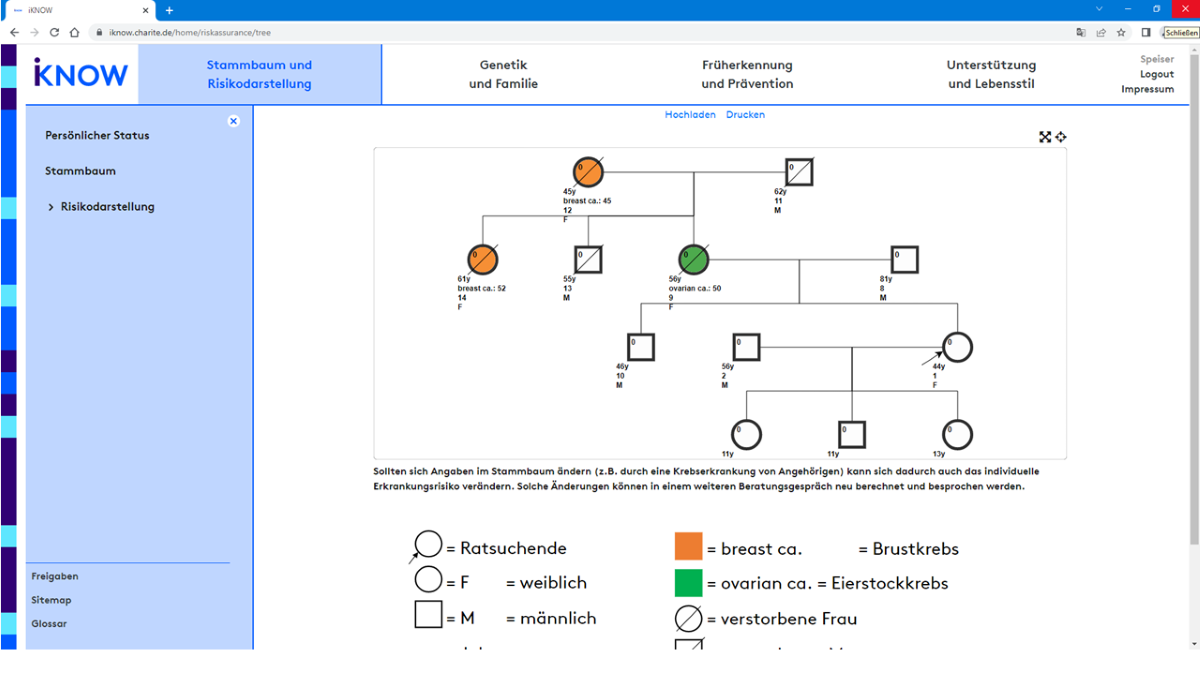
iKNOW counseling tool. Individualized family tree (available in the physicians’ and patients’ version) using BOADICEA [9]. BOADICEA: Breast and Ovarian Analysis of Disease Incidence and Carrier Estimation Algorithm.

**Figure 5:**
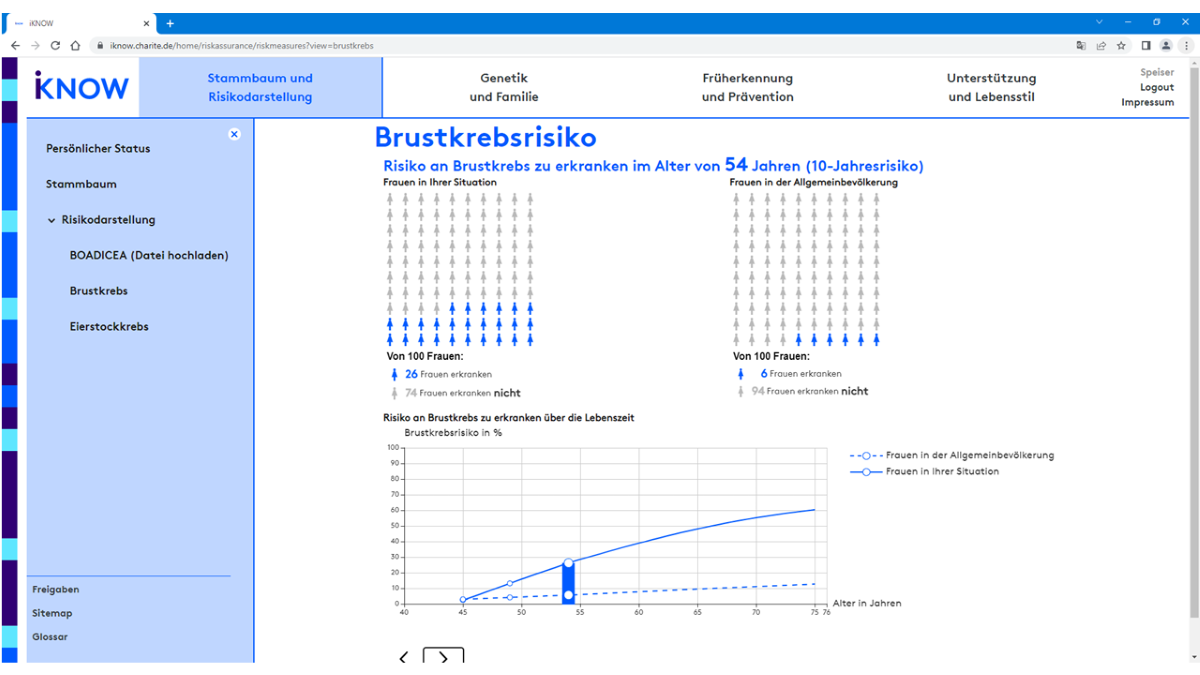
iKNOW counseling tool. Risk visualization for 10-year risk of breast cancer for a carrier of a pathogenic gBRCA1 variant using diagrams and icon arrays. BOADICEA: Breast and Ovarian Analysis of Disease Incidence and Carrier Estimation Algorithm; gBRCA1: germline breast cancer gene 1.

The first level is meant to be used during the counseling session, where physicians use the headings to select the contents to be discussed. Once selected, iKNOW provides level 2 information (ie, headings plus visual representations), so important concepts and relationships can be clarified visually, but the focus of the conversation remains on the doctor-patient interaction rather than on detailed explanatory text. Given the central role of personalized cancer incidence risks in this setting, a particular emphasis of iKNOW’s level 2 information is on *risk visualizations*. First, as the basis of any risk calculation, the counselee’s familial cancer history is visualized in the form of a family tree (Figure 4). In addition, counselees’ personalized risks to get breast and ovarian cancer are displayed for different time intervals based on input from risk calculation algorithms such as BOADICEA [9]. To facilitate the understanding of risks over time, we followed standards of risk communication, recommending diagrams to display changes over time [24,25], and icon arrays to compare risks at clinically relevant points in time (ie, 1-year, 5-year, 10-year, and lifetime risks) [26]. An example of this combined risk visualization for the 1-year risks (top) and 10-year risks (bottom) is provided in Figure 5.

**Figure 6:**
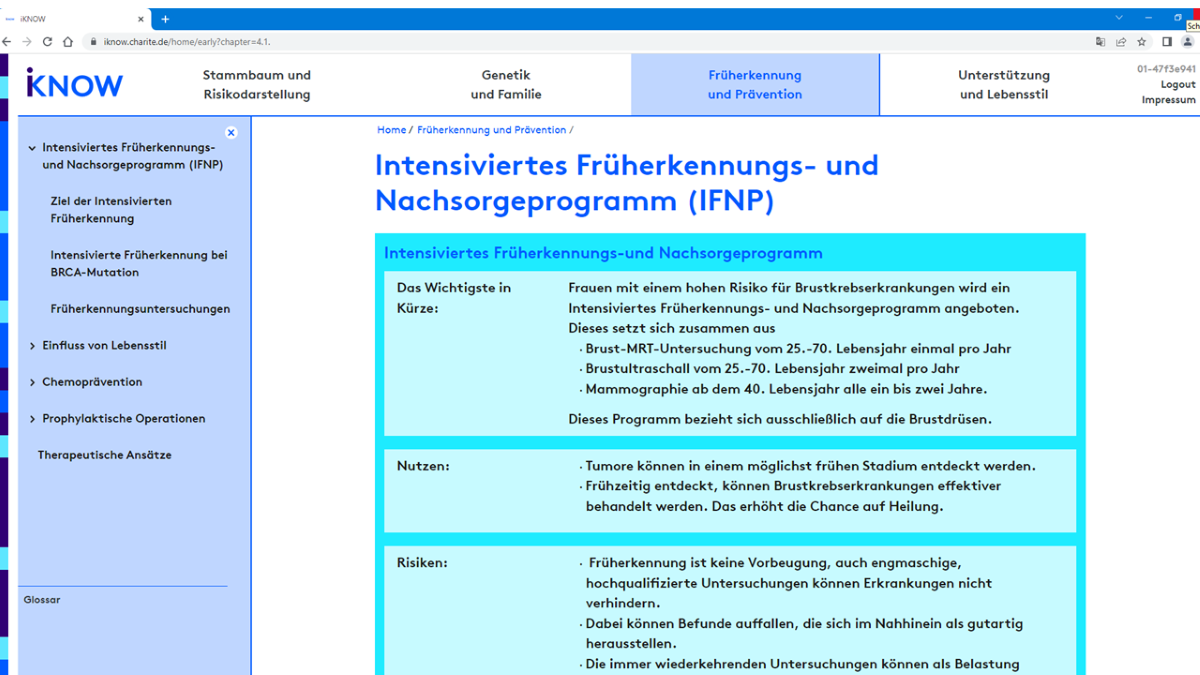
iKNOW counseling tool. To allow patients to look up information after counseling, a concise, text-based summary of counseling contents is provided in addition to the visuals presented during counseling, conveying a takeaway message and a verbal summary of benefits (eg, early tumor detection) and risks (eg, false alarms). BRCA: breast cancer gene; MRT: MRI (magnetic resonance imaging).

After a topic has been discussed, the physician may tick a checkmark to specify if the discussed contents should be available to the patient after the counseling session. When the patient reaccesses iKNOW, she can then see level 3 information (ie, a headings plus visual representations plus explanatory text). The additional easy-to-understand explanatory text in level 3 is provided to augment the tables and visuals and summarize what has been discussed with the physician (Figure 6). The idea is that counselees may use the combination of text and visuals to deepen their understanding of what has been discussed orally during counseling. The level 3 information summarized in iKNOW may also provide patients with the opportunity to share counseling contents with their family members and discuss their situation (ie, individual cancer risks) and ways to cope with it (eg, preventive and treatment options as well as psycho-oncological support programs).

Based on current guidelines for how to format health information, the explanatory text for level 3 information should be written at a fourth-grade reading level using short, active sentences, a verbal style, explanations of technical terms, and links to the cited evidence [27]. In addition to easy-to-understand text design and given the complexity of individual cancer risks, we also developed the following principles to facilitate the understanding of medical and biological technical terms: (1) all technical terms are explained in plain language in the text, (2) definitions are provided via a “mouse-over” feature (ie, when a patient hovers with her mouse over a technical term, the definition appears in a pop-up window), and (3) a glossary is provided that can be accessed via direct link by clicking on any technical term in the texts. To address different preferences for information, we are planning to develop additional versions of these texts in the future so that physicians can provide counselees with different levels of detail and evidence upon request. Also, foreign-language versions may then be implemented.

Finally, also the technical implementation of the counseling tool should be designed for easy access. The iKNOW tool can be accessed via the internet using various devices such as computers, smartphones, or tablets. Its interface design is responsive, that is, it automatically adapts to the screen sizes of the hardware used to access it. For optimal user experience of iKNOW during counseling, however, we recommend a computer screen that is at least 17 inches wide. Patients are advised to use screen sizes of at least 8-10.5 inches for optimal experience at home, but they can access iKNOW also on a smartphone.

### Principle 4: Respecting Counselees’ Data Privacy Rights

In addition to providing the best possible care for patients and counselees with familial cancer risks, genetic counseling raises important ethical and legal questions. These questions relate to data privacy and data management both with respect to the information counselees receive during counseling and the possibility that individuals may be identified based on their genetic makeup. iKNOW considers both aspects, which are covered by the Genetic Diagnostics Act (Gendiagnostikgesetz) and the General Data Protection Regulation (GDPR) in Germany, respectively.

According to the German Genetic Diagnostics Act, a person has the “right *not* to know” and dismiss information about any aspect of their genetic condition. Thus, iKNOW takes a cautious approach to providing information and releases information for each topic separately and only after obtaining consent (via a mouse click) [17,28]. That is, during every step of the counseling process, it is the physicians together with the counselees who decide whether or not a particular piece of information should be discussed. Similarly, if counselees decide that they want to reaccess any aspects of the information they have discussed during counseling, the physicians must actively check a box in iKNOW. Otherwise, the information will be hidden in the counselees’ version.

With respect to data privacy and the protection of counselees’ personal data, iKNOW implements a complex data protection plan: (1) both the physician and the counselee receive a randomly generated access code and password (2-factor authentication); (2) no personal information (eg, names and patient numbers) is processed or saved in iKNOW so that counselees cannot be identified, even if the access code is lost and entered by a third person; alternatively, access to personalized data can and should be restricted to physicians and their counselees to prevent unauthorized access by, for instance, software developers or administrators; (3) should the counselee lose the access code, a new one can be generated only by the physician who is familiar with the patient; and (4) all data entered in iKNOW are stored on protected servers.

### Principle 5: Systematic and Iterative Evaluation With the Users in Mind

Systematic, iterative evaluation based on both quantitative and qualitative methods should be an integral part of any user-centric development process of digital technologies [29]. In iKNOW, prototypes of all features have been pretested formatively with counselees or physicians using both qualitative methods (eg, interviews and observations) and quantitative assessments (eg, usability questionnaires). The evaluation results have been fed back to an interdisciplinary development team (comprising physicians, expert patients, psychologists, ethnographers, programmers, and usability specialists), which decided how to redesign features to better fit user needs. Once saturation of the pretests was reached (ie, no new insights were generated), the iKNOW features were implemented. The completed tool is currently being evaluated summatively in the clinical setting using a randomized controlled trial (RCT) to identify the effects of iKNOW on clinical and psychosocial outcomes and via a qualitative evaluation study to investigate changes in the actual counseling practice due to the iKNOW technology [30].

Specifically, iKNOW is currently tested against the standard counseling in an RCT with 2 parallel groups with a total of 220 counselees or patients randomly assigned to consultations with the iKNOW tool (intervention group) or without (control group). To evaluate iKNOW as a counseling support tool, analyses will focus on accurate risk understanding as the primary end point (ie, group differences between the intervention and control group with respect to the absolute deviation of the counselees’ risk estimates from their calculated risks 6 months after their consultation) and psychosocial burden as the secondary end point (eg, cancer worry and state anxiety). Both outcomes are essential preconditions for shared decision-making.

To capture changes of the counseling practice due to iKNOW, a *qualitative evaluation study* is run alongside the RCT in both study arms, including observations of counseling sessions with and without iKNOW and interviews with both counselees and physicians. This type of evaluation has proven particularly helpful because, unlike their quantitative counterparts, qualitative studies are ideally suited to *capture unpredictable changes* of the counseling practice (eg, due to the COVID-19 pandemic, where iKNOW was suddenly used to support video consultations).

## Outlook and Conclusions

Digital health technologies are playing an increasingly important role in clinical practice. We presented a principled approach to designing digital support interventions for people with hereditary cancer risks, introducing iKNOW as the first German-language digital counseling tool in this area. After successful evaluation, the next step will be to transfer the iKNOW tool to standard care. Also, we are currently using the same principles to build on iKNOW and design a digital tool that supports and integrates information exchange across the entire care pathway of patients with hereditary cancer risks (ie, from community physicians’ offices to specialized clinics and back) [31].

Of course, the specifics of our design concepts are bound by the context of the German health care system and the specific case of hereditary breast and ovarian cancer. Nonetheless, we hope that the general principles we identified will be helpful to guide the development of digital health tools in other fields of personalized medicine and health care systems. Also, rigorous evaluations must show how the concept of iKNOW as a clinical *counseling* support tool compares with the classical notion of clinical *decision* support tools both in terms of clinical and process outcomes and patient-reported outcome measures. In the meanwhile, we hope that the presented design principles serve as a blueprint to motivate the development of more digital innovations and that it will be revised or updated based on the experiences of other researchers with other applications. Ultimately, we hope that a principled approach will help to further advance the field of digital support tools and more quickly realize their potential for fast emerging fields such as personalized medicine.

## Abbreviations

BOADICEA: Breast and Ovarian Analysis of Disease Incidence and Carrier Estimation Algorithm
BRCA1: breast cancer gene 1
BRCA2: breast cancer gene 2
gBRCA: germline breast cancer gene
GC-HBOC: German Consortium of Hereditary Breast and Ovarian Cancer
RCT: randomized controlled trial
